# The Use of Implementation Science Tools to Design, Implement, and Monitor a Community-Based mHealth Intervention for Child Health in the Amazon

**DOI:** 10.3389/fpubh.2020.00411

**Published:** 2020-08-19

**Authors:** Christopher Westgard, W. Oscar Fleming

**Affiliations:** ^1^Department of Maternal and Child Health, University of North Carolina at Chapel Hill Gillings School of Global Public Health, Chapel Hill, NC, United States; ^2^Department of Research, Elementos, Lima, Peru; ^3^National Implementation Research Network, Frank Porter Graham Developmental Institute, University of North Carolina at Chapel Hill, Chapel Hill, NC, United States

**Keywords:** implementation science, implementation research, mHealth, child health, Amazon, Peru, health promotion, active implementation frameworks

## Abstract

It is essential to analyze the local context and implementation components to effectively deliver evidence-based solutions to public health problems. Tools provided by the field of implementation science can guide practitioners through a comprehensive implementation process, making innovations more adaptable, efficient, and sustainable. It is equally important to report on the design and implementation process so others can analyze, replicate, and improve on the progress made from an intervention. The current study reports on the design and implementation of an mHealth intervention to improve child health in the Amazon of Peru. The study aims to provide insight into how an implementation science tool can be used to improve implementation and reporting of an evidence-based intervention in a global health setting.

**Methods:** Implementation of a community-based mHealth intervention is analyzed and reported through the lens of the Active Implementation Frameworks (AIF). The AIF is used to analyze the design, implementation, adaptation, and monitoring of the intervention. The implementation process is categorized in the four stages of implementation. The results of the analysis and subsequent implementation activities are reported.

**Results:** The exploration stage was used to learn about the local context in the Amazonian communities and identify an evidence-based solution to address poor child health. Several potential solutions were combined to create an innovative mHealth tool. During the installation stage, the stakeholders worked together to improve the intervention and plan for implementation through human-centered design. The providers in the field were trained and data was gathered to monitor implementation. During initial implementation stage, electronic tablets were distributed to community health agents and continuous quality improvement activities allowed for rapid improvements to be implemented. The intervention moved on to full implementation stage as acceptance and fidelity approached 100%.

**Conclusion:** The AIF highlighted several potential barriers to implementation that may have been overlooked without the guidance of a science-based implementation tool. Reporting on the implementation process shows how implementation science tools can be used to foresee and address potential threats to successful implementation. The results of this study provide insight into the components of implementation in Amazonian communities, as well as the process of using implementation science tools in any global health setting.

## Introduction

Many public health interventions that have been proven to be effective in controlled settings are not creating the expected impact when replicated in community settings (1–6). There are interventions that have been effective at improving child health and development in low-resource community settings, however, replicating and scaling these interventions have been challenging (7, 8). For example, home visits by health promotors have been shown to be effective, though outcomes vary greatly (9–12). Progress to improve and scale evidence-based interventions to address poor childhood development has been slow, partly due to difficulty adapting interventions to diverse contexts and a lack of reporting on the implementation process conducted by researchers (5, 6, 13).

The implementation process is complex and influenced by diverse factors. Prior to implementing an innovative program in a new context, it is essential to determine if it can be effective and if adaptations are needed to enhance its potential impact. Understanding the context helps to improve the fit of the innovation and implementation strategies, thus improving feasibility, acceptability, and sustainability (1, 14).

Implementation science proposes various theories, models, and frameworks (called tools henceforth) that can be used to improve diffusion of evidence-based interventions, adapt innovations to local contexts, better understand the implementation setting, and evaluate the implementation process (2, 15–18). However, few studies have been conducted that report on the use of the tools in global health settings and the resulting implementation process (13).

The current study reports on the design and implementation of an mHealth intervention to improve child health in the Amazon of Peru. The study aims to provide insight into how an implementation science tool can be used to improve implementation and reporting of an evidence-based intervention in a global health setting. Reporting on the implementation process is expected to show how implementation science tools can be used to foresee and address potential threats to successful implementation. This report addresses the need for critical reflections from practice-based settings to give insight into the barriers and facilitators of effective implementation in community-based settings (3).

## Methods

### Study Procedure

The current study utilizes an implementation science tool to systematically design, implement, monitor, adapt, and report on a community-based mHealth intervention for child health. The study utilizes a systematic method for choosing the most appropriate implementation science tool for the initiative. The tool is used to ensure the key components to effective implementation are considered and supported. The tool is also used to guide reporting of the implementation process to ensure all relevant activities are described here. The implementation process is categorized into four stages of implementation to display the challenges to implementation and the solutions that were provided. The analysis focuses on the use of information gathering to identify and improve an intervention and the implementation process, the implementation outcomes (fidelity, acceptability, adoption) and training for quality improvement. The analysis of the process and outcomes are reported in the results.

### Study Setting

This article describes the implementation of an intervention to improve the impact of community health agent (CHAs) programs on child health and development outcomes. The study took place in the northern Amazon region of Peru, in the department of Loreto. In Loreto, 57% of children under 3 have anemia, 20% under 5 have chronic malnutrition (2018), and infant mortality rate is 30 deaths per 1,000 live births (19–21). Delay in early childhood development was reported to be experienced by 26.7% of children in the region (2017) (22). Many of the illnesses can be mitigated by better practices in the household that lead to better sanitation practices, nutrition, and disease prevention (23, 24). However, caregiver's knowledge of practices to maintain a healthy family are limited as they transition from traditional practices to modern medicine (25–28). To improve health in the communities, the population must understand the causes, consequences, and treatments of poor nutrition and infection (24, 29–31).

In Peru, CHA programs are widely used but greatly fragmented, with each level of government (national, regional, local) operating distinct programs. Although they share the objective of improving maternal and child health, each program has a different system of operations, incentives, supervision, material, etc. While some communities have CHA programs from all three levels of government, others have none. The CHA programs often lack effective job aids to guide health education and data collection.

To address these concerns, Elementos, a Peruvian research organization, used an implementation science tool to guide the process to identify a potential solution, co-create the design, and implement the innovation. The objective was to improve child health and development, by improving the capacity of CHA programs to conduct health promotion and surveillance.

During the pilot study, which is the focus of the current paper, the innovation was tested in a randomized control trial, in 6 communities, with 20 CHAs, serving 230 children. It was provided to established CHA programs for them to use during their regularly scheduled home visits with caregivers of children 0–3 years of age. The communities are only connected by rivers, approximately 6 h by boat (1.5 h by speed boat) from the department capitol of Iquitos. Each community has a population between 500 and 2,000 people. The communities have sporadic cell phone signal and at least 3 h of electricity per day. The study protocol is described in Westgard et al. (32).

### Implementation Science Tool Selection

The first step for the implementation process was to choose the appropriate implementation science tool. A list of potential tools and their level of analysis is included in the online tool, *Dissemination and Implementation Models in Health Research and Practice* (33). The list is extensive with little information on how each tool can be used. Previous knowledge of implementation science and its tools or additional reading is necessary for the list to be meaningful. The authors of the current study utilized their knowledge of implementation science, along with additional study of the various tools, to select the five most promising tools for the project. The five tools were: (1) Active Implementation Frameworks (AIF) (18); (2) Consolidated Framework for Implementation Research (CFIR) (15); (3) The Exploration, Preparation, Implementation, Sustainment Framework (EPIS) (2); (4) Interactive Systems Framework for Dissemination and Implementation (ISF) (34); and (5) the Theoretical Domains Framework (TDF) (35).

To compare the tools and select the most appropriate for the project objectives, the *Theory, Model, and Framework Comparison and Selection Tool* (T-CaST) was used (36, 37). The T-Cast helped the authors systematically think about the strengths of each tool as they relate to each criterion that is important for successful implementation. The criteria were chosen from a list, based on the project's objectives. The tools were scored across the following 7 criteria: the tool includes relevant constructs, provides a step-by-step approach for applying it, provides an explanation for how constructs influence implementation, focuses on relevant implementation outcomes, addresses a relevant analytic level, proposes testable hypotheses, and contributes to an evidence base. Based on the score of each criterion, the authors were able to differentiate the most appropriate tool.

The AIF scored the highest in the evaluation with an average score of 1.57. It was therefore selected to be the tool utilized to guide the implementation research and practice. The average score of each tool is in Table 1.

**Table 1:** T-Cast scores.

**AIF**	**CFIR**	**EPIS**	**ISF**	**TDF**
1.57	1.00	0.86	1.00	0.86

### Active Implementation Frameworks

**Figure 1:**
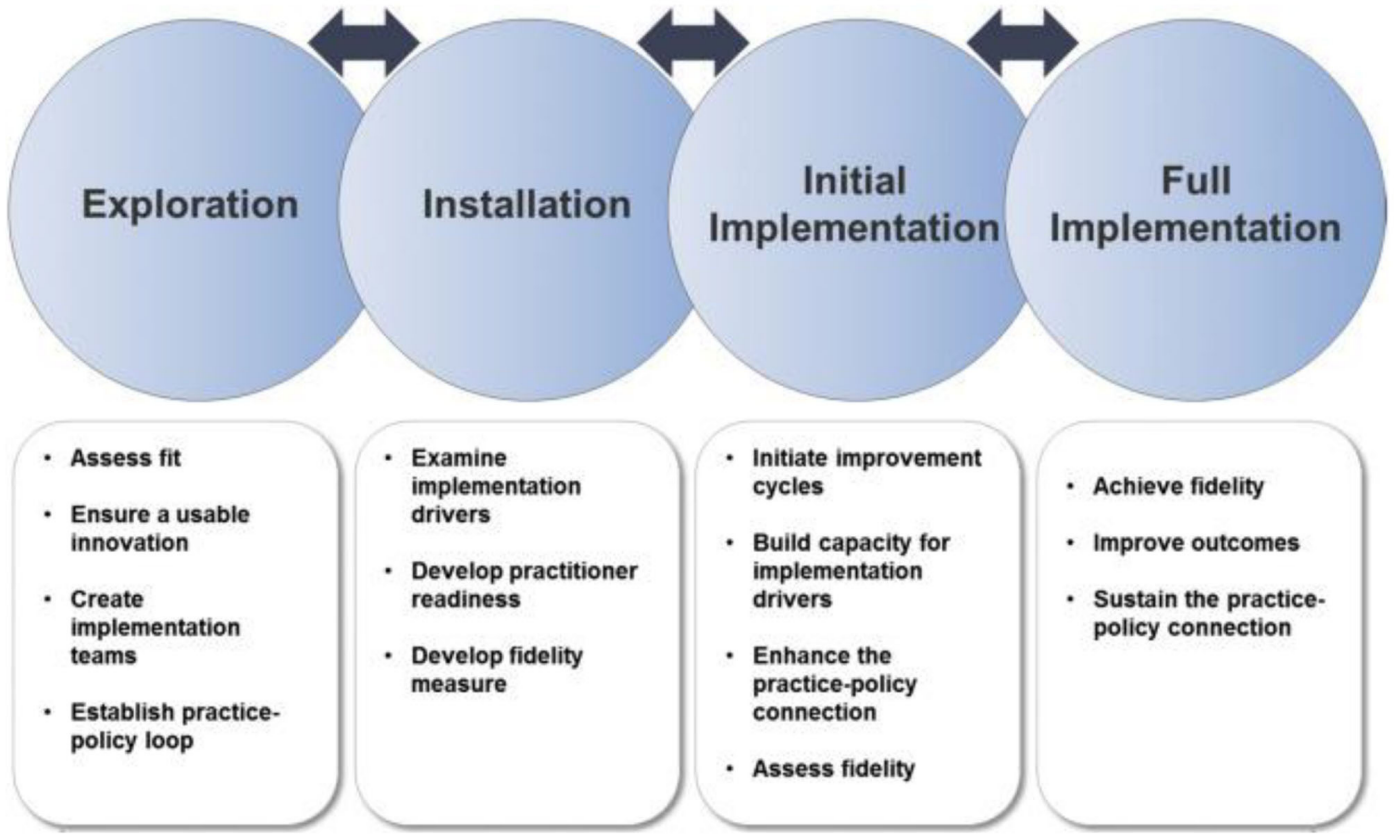
Implementation stages framework.

The AIF is comprised of five distinct frameworks. The 5 Active Implementation Frameworks include; (1) Usable Innovations, (2) Implementation Stages, (3) Implementation Drivers, (4) Implementation Teams, (5) Improvement Cycles. Through the application of the 5 AIFs, users are guided through the stages and key activities for successful implementation, supporting careful consideration of the implementation setting and components of the intervention [see Figure 1; (38, 39)]. The stages of implementation include (1) *Exploration* of the local context and identification of innovations that can create positive change, (2) *Installation* of the capacity and resources needed to introduce, improve and sustain an innovation; (3) *Initial implementation*, during which performance data is used to rapidly improve both the innovation and implementation supports and strategies; and (4) *Full Implementation*, where high quality implementation and program outcomes are realized and sustaining performance is a core focus.

The current study reports on the process used to move from exploration to full implementation of the innovation in the communities. The Implementation Stages Framework, of the AIF, is used to organize the description of the evolving implementation process. The other components of the AIF are presented within the various stages of implementation (18, 40). The implementation process during each stage is described with the aim of reporting on the key components addressed to accomplish implementation. Key challenges that arose and the decisions that were taken to address them are presented.

## Results

The results of the analysis of the implementation process is presented in the sections below, representing the four stages of implementation. The authors explore the critical components of project implementation as they relate to each stage of implementation.

### Exploration Stage: Identifying Challenges and Solutions

The exploration stage involved understanding the needs of the communities, identifying evidence-based practices that can address their needs, and determining the right fit between potential solutions and the local context. The work done during this stage improved the chances for success of the program by checking to ensure the local population wanted the intervention and believed it could work within their reality (41, 42).

The research team conducted formative research in the communities to better understand the needs and priorities of the families. This involved the following activities:

- Interviewed regional health directors, program coordinators of municipalities, and community leaders to learn about their most pressing health issues, their priorities, and key barriers to progress. A common consensus among all stakeholders was the problem of child malnutrition and poor early childhood development.
- Conducted a social determinants study to better understand the drivers of poor child development in the communities. The study found that poor sanitation and nutritional practices were associated with an increase in developmental delay, and contact with a CHAs was associated with a decrease in developmental delay (22).
- Conducted a study to better understand the barriers to utilization of local health services for maternal and child health. The study identified key reasons why some mothers do not attend health-checkups for their child nor give micronutrient supplements. Long wait times, closures, and a mistrust of health center personal were among the top reasons (26).
- Conducted a performance evaluation of CHAs in the communities. Through observations of home visits by CHAs, the study found that many CHAs lacked the capacity and material to transmit the knowledge needed by the caregivers to conduct healthy maternal and child health practices (43).

The studies and informant interviews identified poor health behaviors within the household as a key driver for child health and development. Unhealthy behaviors that were taking place included: drinking untreated water; early cessation of breast feeding; poor diet; poor handwashing practices; unsanitary toilets; and low use of nutrient supplements and deworming medication (22, 44, 45). Caregivers often lacked a good understanding of the causes, consequences, and treatment of common childhood illnesses. The local stakeholders agreed that health promotion and education were greatly needed, and that CHAs are a strong potential mechanism to provide that service. Evidence from the studies suggested that improved performance of CHAs could improve the knowledge and practices of caregivers, and thus improve the child health and development outcomes.

Following the decision to focus on CHAs to address poor child development, additional research was conducted on the policies and operations of the CHA programs in the region. The research team conducted interviews with representatives of CHA programs at the national, regional, and local level. It was soon discovered that representatives at each level operated a distinct CHA program. The programs share the objective of improving maternal and child health, however, each program has a different system of operations, incentives, supervision, recruitment, and material. While some communities have CHAs from all three levels of government, others have none.

The research team studied the operations of the CHA programs to better understand how an intervention could be designed and implemented to improve CHA performance and impact. The research revealed several of the barriers expressed above: CHAs struggle to remember and transmit the knowledge needed to teach caregivers, they lack direction to choose which health messages should be shared at each home visit, and they lack material to help transmit the information. Additionally, there is a lack of supervision and any control of the quality of the home visits. Fidelity of the CHA program suffers from a lack of a responsive supervisory system. The supervisors and representatives of the health centers and Ministry of Health have little way of determining if home visits are being conducted as intended.

A landscape analysis was conducted to identify evidence-based interventions with the potential to improve CHA performance in the Amazonian communities. Potential interventions were identified by reading scientific literature, expert interviews, and assessing the tools shared by CHW CENTRAL^1^ and the Community Health Worker Assessment and Improvement Matrix (46). A list of potential interventions was evaluated through a policy analysis to determine which best satisfied the selection criteria and showed most promise to be successful in the low-resource community setting. The search for potential interventions and the comparison process was dynamic, with new interventions being added and deleted over several months. The analysis revealed that multiple interventions had the potential to create positive impact in the CHA setting. By utilizing mobile information and communication technology (ICT), several of the intervention components could be combined into one innovative intervention.

Several studies have shown that mobile ICTs can improve the performance of CHAs in their ability to perform health promotion, collect and report timely information regarding family health, provide health services such as vaccines, and refer families to appropriate local health services (3, 47–53). Additionally, when a mobile ICT tools are used by a CHA, the device can increase the confidence the caregivers have in the messages being transmitted and increase the confidence the CHAs have in their own work (47, 48, 51, 52, 54–56). Through implementation science, innovations in mobile ICTs and strategies for child health and development can be extended to low resource settings to empower local actors and spread the benefits of advancements in technology (3, 50).

The evidence-based interventions that showed promise to improve CHA performance included: conducting surveillance of maternal and child health indicators with a mHealth tool, utilizing animated videos to deliver health messages to encourage behavior change, harnessing health behavior theory for the creation of health messages, and improve self-efficacy of CHAs by providing dynamic tool (24, 30, 31, 47–49, 51–53, 55, 57, 58). The intervention components were combined to create an innovative tool that supports CHA programs.

The innovative tool was titled, *The Child Health Education and Surveillance Tool Application* (The CHEST App). A video of the App can be viewed online (59). The CHEST App is an android-based application downloaded onto an electronic tablet. The CHEST App provides the steps for the CHA to follow to conduct an effective home visit with caregivers. It was designed to improve the capacity of CHAs to transmit knowledge of healthy child-rearing practices and conduct disease surveillance. The CHEST App provides the following functions: (1) collect child health indicators at the household level and upload the data to the server; (2) select appropriate health messages to deliver during the home visit based on the age of the child; (3) share animated videos, images, and statements that reinforce the health messages; (4) calculate and display the anthropometric and nutritional status of children; and (5) organize the case load of children and maintain schedules for home visits and health check-ups (32).

A full description of the intervention can be seen in the study protocol that was published in 2019 (32). The theory of change for the intervention is displayed in Appendix 1 in Supplementary Material.

Once the CHEST App intervention was defined, it needed to be assessed to determine fit and feasibility for success in the local context. The intervention was assessed alongside the implementation setting to determine the probability of success. The exercise was supported by the Hexagon Exploration Tool, of the AIF. The Hexagon Exploration Tool guides selection and evaluation of potential interventions for an implementation setting by promoting the consideration of key program and implementation site indicators [Appendix 2 in Supplementary Material (63)]. The CHEST App was assessed with the Hexagon Exploration Tool by considering the six key components for successful implementation, as displayed in Table 2. The exercise confirmed the potential for success of the intervention in the communities and promoted further consideration of important components of implementation.

**Table 2:** The hexagon exploration tool assessment of the CHEST App.

**Program indicators**	
Evidence	Multiple studies have shown that mHealth tools can improve CHA performance in similar low-resource community settings, including health education with videos and digital surveillance tools (47–49, 51–55)
Usability	Previous studies and stake-holder interviews suggested that the technology could be used in the local context. Additionally, the acceptability and usability of the CHEST App was confirmed through informal interviews with the local populations. The CHAs and local supervisors expressed their preferences for how the tool should be designed to fit the needs of their program.
Supports	Elementos had the resources to design and implement the CHEST App thanks to funding from Grand Challenges Canada, Saving Brains grant (60). Elementos created the capacity to conduct the project by hiring a multidisciplinary team of specialists; an implementation scientist, nutritionist, anthropologist, community psychologist, epidemiologist, nurse, communicator, and software engineer. Together they developed the material (a guidebook of health messages and animated videos), the App, and the implementation protocol. The CHEST App was developed from open source code from OpenSRP, (61) which includes code and forums for support. Technical assistance and development was also provided by the UNC CHAI Core team (62)
**Implementing site indicators**	
Capacity to implement	The communities have established CHA programs that have the capacity and interest to receive and integrate the CHEST App into their normal activities. The CHA programs are supported by funding from their Municipality, which includes pay for a program supervisor. Additional implementation support was provided by Elementos by providing an implementation team that visits the communities for continuous training and support for 1 year.
Fit with current initiatives	The tool was created to integrate within the established CHA programs with minimal interruption of their current activities. The tool was expected to improve the ease and effectiveness of their current initiatives.
Need	Multiple studies by the research team identified the need; reflected by the high rates of malnutrition, misunderstanding of health topics by caregivers, and poor performance of CHAs.

### Installation Stage: Preparations to Initiate New Program

After exploration, efforts shifted to preparing for implementation. An implementation team at Elementos was created to assist the actors in the field. The implementation team created the initial plan for implementation, prepared the local actors, and readied the tools, and material for the intervention. They conducted the training, monitoring, and quality improvement cycles. The team consisted of an implementation scientist, nurse, nutritionist, and anthropologist. Before going to the field to prepare the local actors, the implementation team was trained on the use of the CHEST App, how to coach the CHAs, how to conduct an effective home visit with the tool, and how to identify and report challenges experienced by the CHAs.

The CHEST App was developed throughout the 6 months of the installation phase. A prototype was needed to show the local actors what the intervention would look like. However, the final form of the App was unknown at the beginning of development because the design needed the input from the end-users and the implementation team needed to further understand the workflow of the CHAs. The multidisciplinary team designed and created the App, the health messages and images, and animated videos to include in the App.

To prepare for implementation, the team needed to determine where and when implementation would take place. To determine the location of the pilot, meetings were conducted with directors of CHA programs at the 3 levels of government (national, regional, and local) to present the CHEST App, document the system of operations of each CHA program, and assess interest in receiving the intervention. The CHA program coordinated by the Regional Ministry of Health has little formal structure or supervision. The CHAs conduct occasional campaigns (such as malaria prevention) and some conduct home visits as their own independent initiative. The CHAs did not receive incentives or regular supervision. Their home visits are thus infrequent and unpredictable, making their program a poor fit to receive the CHEST App. At the national level, the CHA program, “Cuna Mas,” conducts home visits in areas of extreme poverty (64). The CHAs receive a modest stipend for their work, close supervision, and continuous training. Based on the structure of the program, they were an excellent fit to receive the CHEST App. The directors of the program were excited to receive the CHEST App. However, working with the national government provided difficult. Significant turnover of staff in the Ministry delayed communications and the formulation of a formal agreement. Ultimately, the team was not able to formulate a formal agreement with the ministry in time to implement. They decided to implement the intervention with the local government CHA programs. The district-level municipalities operate their own CHA program, which vary by districts. All include home visits with children under 4, an incentive package (stipend or gift baskets), and a program supervision. The 5 districts that were approached by the implementation team (Amazon, Indiana, Las Amazonas, Fernando Lores, and Punchana) were enthusiastic to receive the CHEST App intervention. They established communications with each district and began learning about the specific activities of the CHA program in each district.

To determine when implementation should take place, the implementation process had to adapt to the political activities in each district. The districts had recently conducted elections, so their new representatives were adjusting programs and budgets, including the CHA program. Many were replacing CHAs and supervisors with their own contacts, freezing the program until a new budget could be released, or changing specific activities of the CHAs. Therefore, installation and initial implementation had to be delayed for the programs to stabilize. Continuous communication with the program coordinators and policy makers in the districts made it possible to continue to improve the intervention and implementation process during the delay. Although this created delay for the start date of the CHEST App intervention, it helped troubleshoot and avoid several potential issues that may have arose.

In each community, leaders were selected to head the initiative in their group. The program supervisor was included as a leader and the supervisor choose one CHA to join the leadership. The supervisor was the primary person of contact in each community. The CHA leader helped lead group discussions, trainings, and share the opinions of the CHAs to the implementation team. The CHA leaders also played an important role in setting the general mood of the group. When the leader decided to accept the intervention and dedicate themselves to learning the new skills, the rest of the group followed even if they were initially hesitant.

To prepare for the evaluation of the intervention and to adjust the program to the local setting, extensive data collection was conducted. The data provided a baseline for adaptive monitoring, evaluation, and learning. Process data was collected on the procedures of the CHAs and supervisors to be able to monitor changes that may occur after implementation of the intervention. The indicators included the number of home visits conducted, number of children visited by each CHA, the reporting procedures of the CHA and supervisors, the health indicators they reported, time delay for the indicators to be reported to the health authorities, and acceptability expressed by all stake-holders. This information allowed the implementation team to later assess acceptability, adoption, and fidelity of the new intervention. This helped determine if doses and quality are changing overtime and identify opportunities to adapt the intervention to achieve greater effectiveness.

An assessment form was created for the implementation team to measure acceptability of the intervention. Acceptability was measured by interviewing the CHAs, supervisors, and caregivers. During each visit to the communities by the implementation team, an interview was conducted with one of each actor. The implementation team filled out the Acceptability Assessment Form with each. The questions pertained to what they liked and disliked about the intervention and suggestions to improve it.

The CHEST App includes a method to track adoption of the tool into the CHA program. The CHA records child health indicators with the App during the home visits. The supervisor connects the tablet to a wifi hotspot and uploads the data from the App to the server. The server can be accessed by the supervisor, health center personal, municipality, and implementation team. They can see if the CHAs are conducting the appropriate number of home visits with the tool and collecting the required information. The supervisor was trained to upload the data from the tablets and interpret the data to determine if the frequency of home visits by the CHAs matches what is expected of them. In this way, adoption can be tracked by all parties.

Fidelity of the intervention was accessed by the supervisor through observations of home visits by the CHAs. An assessment form was created by the implementation team and supervisors to assess fidelity. The fidelity assessment form included the steps needed for a quality home visit and scoring system for each step. The supervisor kept a record of fidelity scores for each CHA and scheduled trainings with the CHAs based on their scores.

Data was collected on the intermediary/mediator variables to test the theory of change of the intervention. The information was gathered from household surveys conducted by the implementation team. The intermediary variables included; performance of CHAs, knowledge of caregivers, childrearing practices, and use of health services. Testing for change along each step of the theory of change helped to determine opportunities to adjust and improve the intervention or implementation process. Data was also collected on the primary outcome indicators; hemoglobin levels, anthropometrics to estimate malnutrition, and early childhood development scores. The data was collected to determine the effective size of the intervention and report the implementation outcomes.

The CHAs and program supervisors were trained on how to use the CHEST App to support their work. The training was designed to teach them how to operate the tablet and application, how to use the tool to improve the interaction with caregivers during home visits, and how to use the information gathered by the tablet to improve their impact. The training lasted 3 days. The first day was focused solely on the use of the App. The implementation team sat down with groups of CHAs to show them how to use the App. Then, the CHAs spent the day practicing, working in groups to help each other resolve problems and remember the steps. The CHA leaders were the first to answer questions from the others before a member of the implementation team stepped in to help. The second day of training included simulations of home visits. One CHA conducted the home visits with the CHEST App while another CHA pretended to be a caregiver in her home. The implementation team conducted several simulations for others to watch to show how the home visits could be more dynamic with the use of the tool. The CHAs mimicked the behaviors of the implementation team and greatly improved how they conduct home visits. The third day of training was one-on-one with a member of the implementation team and each CHA. The member of the implementation team accompanied the CHA on a home visit with a caregiver in their community. The implementation team member gave advice to the CHA after the home visit on how it could be improved to better transmit the knowledge displayed in the app.

At times, the supervisor joined home visits with the CHA and implementation team member. At this time, the supervisor was trained in how to assess fidelity with the Fidelity Assessment Form. Before and after the home visits, the implementation team showed the supervisor how to score the home visit on the Fidelity Assessment Form, creating a common standard for a quality home visit. Through the conversations and observations of quality home visits, the supervisor learned how the intervention is intended to be delivered.

The CHAs were initially nervous to use the new tool, albeit excited by the novelty. Many of the CHAs had never used a touch-screen device before. At the end of the 3-day training, all CHAs were able to conduct a home visit on their own with the CHEST App. However, ~20% of the CHA needed additional practice with the App to become faster and more comfortable. A total of 20 CHAs were training, in groups with an average size of 6 CHAs.

During the workshops, the implementation team worked together with the CHAs and supervisors to identify opportunities to further adapt the CHEST App to match their needs. The team conducted human centered design exercises to surface challenges they anticipated from using the new tool and elicit suggestions for how it can be improved. The research team noted the difficulties and suggestions that were expressed and took them back to the developers so they could make quick, incremental improvements. For example, the language used in the App needed to be updated to include more localized terminology. Also, the images used to indicate if a child has chronic or acute malnutrition were removed because they caused confusion. The option in the App to record the child's ID number was made optional because we learned some children do not have a government-issued ID.

### Initial Implementation Stage: Rapid-Cycle Problem Solving

The initial implementation stage began by distributing the tablets with the CHEST App to the CHAs and supporting the integration of the new tool into their normal activities. A total of 20 CHAs in five communities received a tablet. The CHAs immediately began using the tool to help choose which child to visit and guide them through their home visits. They collected data on child health indicators while in the homes and coordinated with the implementation team to upload the data to the server.

This stage of implementation was about testing and improving the functionality of the CHEST App and the implementation process. The implementation team continued to work with the CHAs to conduct improvement cycles on the intervention, further train the CHAs in the use of the CHEST App, support the program supervisors on downloading the data and making data-based decisions, and communicate with the authorities of the municipalities to share the advancements and value of the CHEST App for their program.

The CHA leaders agreed to meet with the CHAs that were struggling to use the App comfortably. Initially, all CHAs in each community met 1–3 times per week to practice using the App. The CHA leaders and supervisor organized the meetings and assisted those that needed help. The meetings became less frequent as they mastered the new tool.

Members of the implementation team from Elementos visited each community bi-weekly during the first 2 months following implementation, and then once a month thereafter during the first year of implementation. The CHA leaders played an important role during the meetings with the implementation team. They voiced the concerns they had about the tool, requested changes, and gave feedback about the general mood of the CHAs in using the tool. The mood was very positive, as the CHAs liked the new tool and the prestige it gave them when they visited the homes.

The families that receive visits from a CHA with the CHEST App were the ultimate recipients of the intervention. Their experience with the CHAs changed due to the new tool. They now have the opportunity to see the health status of their child displayed in the app with stop-light indicators (red or blue), view images and videos that explain topics of health and development, and hear the CHA give guided messages to promote behavior change.

To measure the effectiveness of the CHEST App intervention, implementation outcomes were tracked and evaluated throughout implementation. The implementation outcomes represented how well the intervention was delivered and received. The outcomes that were tracked included adoption, fidelity, and acceptability.

The implementation team and local authorities used the CHEST App as a decision-support data system to assess adoption. Adoption was accessed by analyzing the number of children the CHAs visited and the number of home visits conducted per month. The number of home visits per month was consistently rising or staying consistent (depending on the community), over the first 4 months of implementation. At month 5 of implementation, the implementation team noticed a sharp drop in number of children visited with the CHEST App in a community. The change signaled a reduction in adoption of the intervention and the need for the implementation team to visit the community to troubleshoot the situation. The team found that several of the CHAs were released from the CHA program due to budget cuts. The team worked with the supervisor and municipality to adapt their program to work with fewer CHAs, prioritizing the children with poor nutrition status, and visiting the healthy children less frequently. This allowed the CHAs that remained to continue to visit all the children in the program. Identifying the problem was possible due to the integrated mechanism in the CHEST App to monitor use of the tool.

The fidelity assessments conducted by the supervisors provided the information needed for targeted training and quality improvements. By observing random home visits with the CHAs, the supervisor identified which CHAs were having problems conducting the home visits as intended. The CHAs that scored poorly on parts of the Fidelity Assessment Form received support from the supervisor and CHA leader to improve their performance on those specific steps of the home visit. The assessment allowed the supervisor to identify which aspects of the home visit were not being delivered with fidelity and focus on those aspects during the on-going training.

The information gathered during the fidelity assessments also improved quality improvement efforts. The supervisor and implementation team found that many CHAs were having trouble remembering to gather child health indicators during the visit. They conducted the educational portion without conducting the surveillance portion of the home visit. With this information, the team made adjustments to the CHEST App, making it required to click through the surveillance section of the App before advancing to the educational section. This proved to be effective at ensuring the surveillance was conducted and that the intervention maintained high fidelity.

The implementation team evaluated acceptability of the intervention immediately after implementation and during the following months. The information recorded in the Acceptability Assessment Form provided valuable information to continuously improve the quality of the intervention. Overall acceptability of the intervention increased over time. Most of the suggestions for improvement occurred during the first 4 weeks of implementation. After adjusting the program based on their suggestions, acceptance, and positive feedback were expressed by all CHAs and supervisors. Evaluation of acceptability by the caregivers revealed that the families found the home visits to be more appealing with the CHEST App. Caregivers, children, and other family members became more interested and attentive during the home visit than before. The CHEST App made the caregivers feel more confident in the information the CHA presented and could more easily understand the messages.

The suggestions gathered during the acceptability assessments provided opportunities to improve the quality of the intervention. The team synthesized the requests for changes and adjusted the CHEST App when appropriate. A change was made to the App because some communities were not able to upload the data from the tablet to the server due to insecure assess to cell phone data. The developers added to the CHEST App the ability to transfer data directly from the tablet to a local computer with a cable. By adding a direct transfer function, the program coordinators were able to extract the data as they needed, without cell phone signal or direct assistance from the implementation team. Additionally, a function to erase a case/child from the caseload was added to the CHEST App interface. The CHAs expressed the need for the function due to children frequently aging out of their program or moving away. The CHAs needed the ability to delete cases without the assistance of the implementation team. The solution seemed obvious once the CHAs explained the need, however, the problem did not occur to the design team until then.

### Full Implementation Stage: Program Integration

Once a high level of acceptability, adoption, and fidelity were reached and maintained, the program began the full implementation stage. After 10 months of implementation support, the intervention was operating with 100% adoption across all active CHA programs involved in the pilot. When a CHA conducted a home visit, they used their CHEST App. Also, acceptability and fidelity were high, and supervisors continued fidelity checks and quality improvement efforts without outside support. One community canceled their CHA program, and thus stopped using the CHEST App. The municipality canceled support for the program due to budget restrictions. They anticipate re-activating the program in the coming months. This is an important detail when assessing sustainability of the intervention when implemented at the district level.

After 10 months, the research team ended their monthly visits to the communities. The CHAs and supervisors did not need ongoing training outside of their own local support. Elementos was able to scale-back resources (staff and travel expenses) invested to support the CHEST App program on the ground. However, Elementos was not yet able to stop all involvement in the programs. The supervisors in three of the communities were not yet able to upload, download, organize, and interpret the data obtained with the CHEST App. A member of the implementation team continued communication with the supervisors of each program to assist with the task of data management. On a monthly basis, the supervisors connected the tablets to a cell phone hot spot or directly to a computer to upload the data to the server. They signaled to the team at Elementos that new data was uploaded. At Elementos, the data was then downloaded and organized in a user-friendly report and sent back to the supervisors. The supervisors then shared the report with the municipality and local health post.

To determine if the CHEST App program (intervention and implementation process) is cost-effective and should be sustained and scaled, an evaluation of the process and impact is needed. Follow-up surveys were planned to be conducted and compared to baseline to determine impact after 12 months of operation. The follow-up surveys were delayed due to the COVID-19 pandemic, still pending at the time of writing this manuscript.

Assessment will include measurement of the performance of the CHAs, knowledge evaluations, and surveys of household practices to measure the impact of the CHEST App on CHAs and caregivers. The improvements in knowledge and practices of caregivers are expected to reduce anemia, reduce chronic malnutrition, and increase early childhood development scores.

Sustainability of the intervention has been measured by tracking the adoption and fidelity scores over time. Both adoption and fidelity were high during the first phase of implementation and has maintained after external support from the research team was withdrawn. Sustainability will be tracked for an additional year to ensure the intervention can be further maintained before scaling. An important component to assess sustainability of the intervention is the cost. The primary expense of the CHEST App intervention is the cost of the tablets. For the pilot project, the tablets were provided by Elementos. The municipalities committed to buying new tablets for the intervention to scale to additional communities in their district and to replace old tablets as they become unusable. Their commitment to dedicate sufficient budget to the CHA program to buy tablets is necessary for the intervention to be sustained. Therefore, sustainability of the program is determined by state actors, and not outside support or funding. The program is expected to be continued as long as child health and development remain a top priority.

The CHA program with the CHEST App is expected to be scaled to additional districts and regions of Peru once sustainability is confirmed. The intervention and implementation process were created so they can be replicated and expanded without a decrease in impact (voltage drop) (65). Training CHAs in new communities can be done with trainers of relatively low expertise. The CHA leaders of past intervention communities can take a lead role in training new communities. The data support can be conducted by a central supporting agency, such as the regional ministry of health or a non-governmental organization. Each municipality can manage the supervision and evaluation of adoption and fidelity. Therefore, the program can replicate with little additional cost and demand for outside support.

The educational material that is included in the CHEST App was created to match the reality of the Amazon region. The food sources, infections, sanitation challenges, etc., matches the experience of Amazonian communities. To scale the program, the educational material will need to be adapted to match the diverse contexts in Peru, such as the high mountains and coastal plains, and include messages in local languages. With the CHEST App, modifying the material to match the local reality is feasible and economical. Once the material is developed and translated, it can be uploaded to the tablets remotely. Additionally, new educational material can be added to the CHEST App as the program advances or to match diverse health challenges that arise. The updates can be distributed without purchase or deliver of new material, only adjust the app's code and connect the tablets.

## Discussion

This is the first study to examine the use of the AIF to analyze and report on the implementation process of a global health intervention for child health and development. By reporting on the process, the reader can learn about the implementation context in the Amazon of Peru and how the tool can be applied to analytically assess key components of implementation. The AIF guided the research team to focus on important components of implementation, thus further dedicating resources and analytical consideration during implementation to increase probability of successful of the intervention. The key components of the implementation process included information gathering to conduct improvement cycles, the implementation outcomes (fidelity, acceptability, adoption) to monitor progress and sustainability, and training for continuous quality improvement. Analyzing the various components gave great insight into the behavior of the participants and local system. Understanding the perspectives and behaviors of the providers, end users, and program coordinators on the ground is a valuable part of the implementation science approach, and essential to create long-lasting behavior change (66).

The CHEST App innovation and the implementation strategy went through several adaptations to better fit with the local context. The implementation science approach was extremely beneficial to guide the multiple design iterations and rapid-cycle problem solving. The results were greatly improved promotional material, app design, and implementation process.

It is important that researchers specify and report on the process used to design, adapt, and implement an intervention (5). Details of the implementation process are needed for others to evaluate, replicate, improve, and scale the intervention (6, 13). This study reports on the implementation process and key components that were assessed during the design and implementation of the intervention. The Consolidated Advice on Reporting ECD guidelines (C.A.R.E guidelines) describe which implementation components should be reporting when conducting implementation research on early childhood development interventions (13). This study reports on those components, including previous evidence of intervention, rational, context of implementation, description of recipients, adaptations that occurred, personnel, methods to assess fidelity, and others. An additional study will be published following the final evaluation of the CHEST App intervention that reports on process and clinical.

## Conclusions

The study contributes to the knowledge base by demonstrating how an implementation tool can be applied in practice in global health. The scientific community has indicated the need for greater reporting on the delivery of public health interventions, especially those in global health (1–6). Activities conducted during the design, implementation, and evaluation of an intervention should be reported so the scientific community can learn what works and what does not. This study provides information on the implementation of a child health and development intervention in a community-based setting. The CHEST App intervention was analyzed and reported through the lens of the AIF. The AIF assisted the research team to consider components of implementation that are often neglected, such as choosing the right solutions that fits local context, information gathering for data driven decision making and adaptations, and monitoring implementation outcomes. The analysis and activities that took place during each stage of implementation are described so others implementing a similar intervention can reflect on the experience and improve their own implementation process. This report contributes to the pool of knowledge needed to improve impact and scale of global health, community-based interventions.

## Data Availability Statement

Publicly available datasets were analyzed in this study. This data can be found here: https://figshare.com/articles/Indiana_Resultados_xlsx/12056100/1.

## Ethics Statement

The investigation was approved by the Institutional Ethics Committee of the National Hospital San Bartolomé in Peru on November 8, 2018 (Exp. Number 15 463–18, Oficio N. 0744–2018- OADI- HONADOMANI- SB). The patients/participants provided their written informed consent to participate in this study.

## Author Contributions

CW was involved in the design, implementation, data collection, data analysis, and writing of the manuscript. WF was involved in the analysis and writing of the manuscript. All authors contributed to the article and approved the submitted version.

## Conflict of Interest

CW was employed by the company Elementos. The remaining author declares that the research was conducted in the absence of any commercial or financial relationships that could be construed as a potential conflict of interest.

## Footnotes

^1^Tools | CHW Central. Available online at: https://www.chwcentral.org/resources-types/tools (accessed December 18, 2019).

## Supplementary Material

Appendix 1 Theory of Change of CHEST App.

Appendix 2 The Hexagon Exploration Tool.
