# Supplementary figures and images for: The Use of Implementation Science Tools to Design, Implement, and Monitor a Community-Based mHealth Intervention for Child Health in the Amazon
Source: Front Public Health. 2020 Aug 19;8:411. doi: 10.3389/fpubh.2020.00411 (PMC7466738; doi:10.3389/fpubh.2020.00411)

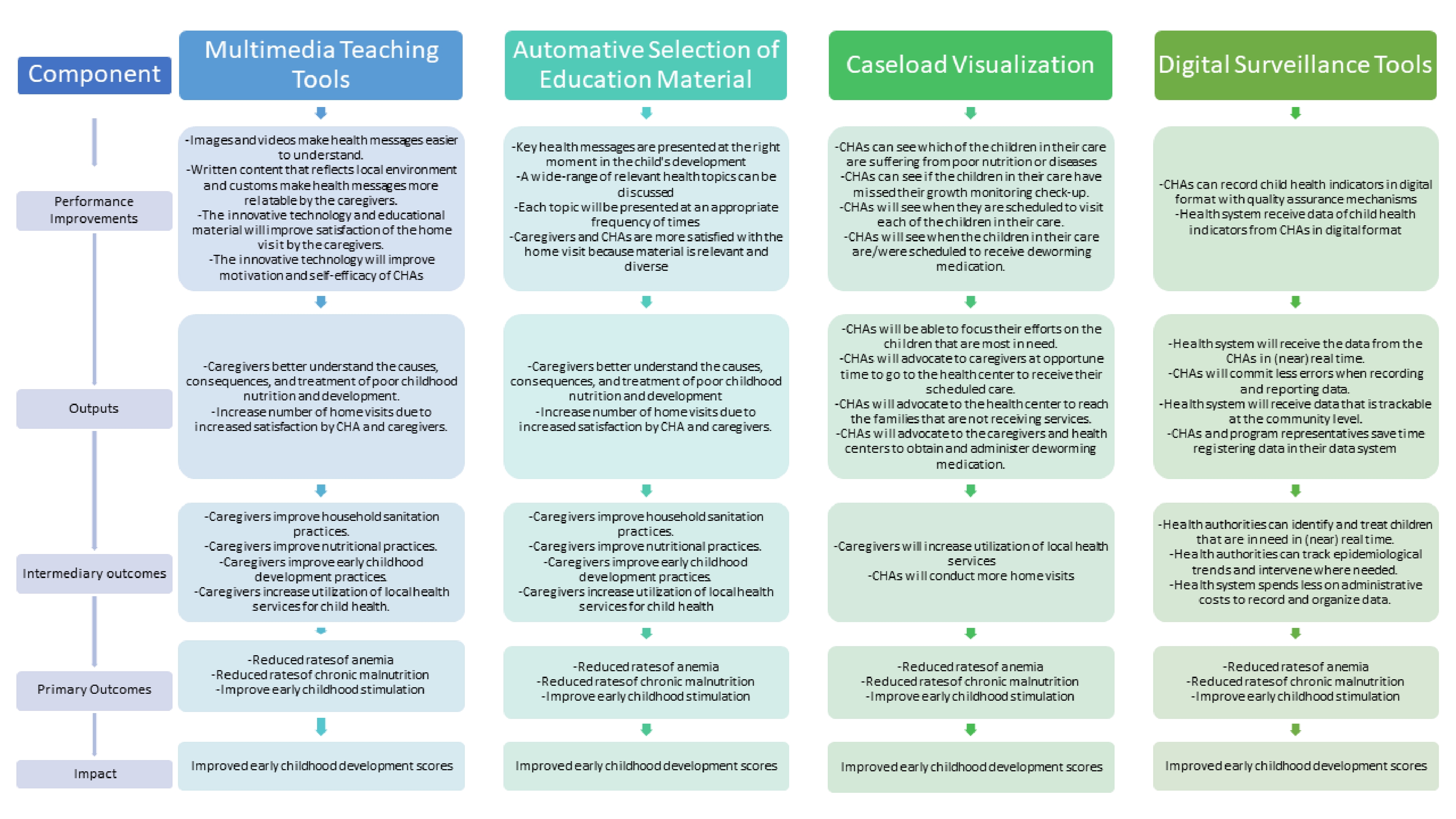

Supplement: Appendix 1 — Theory of Change of CHEST App. [file Image_1.JPEG]

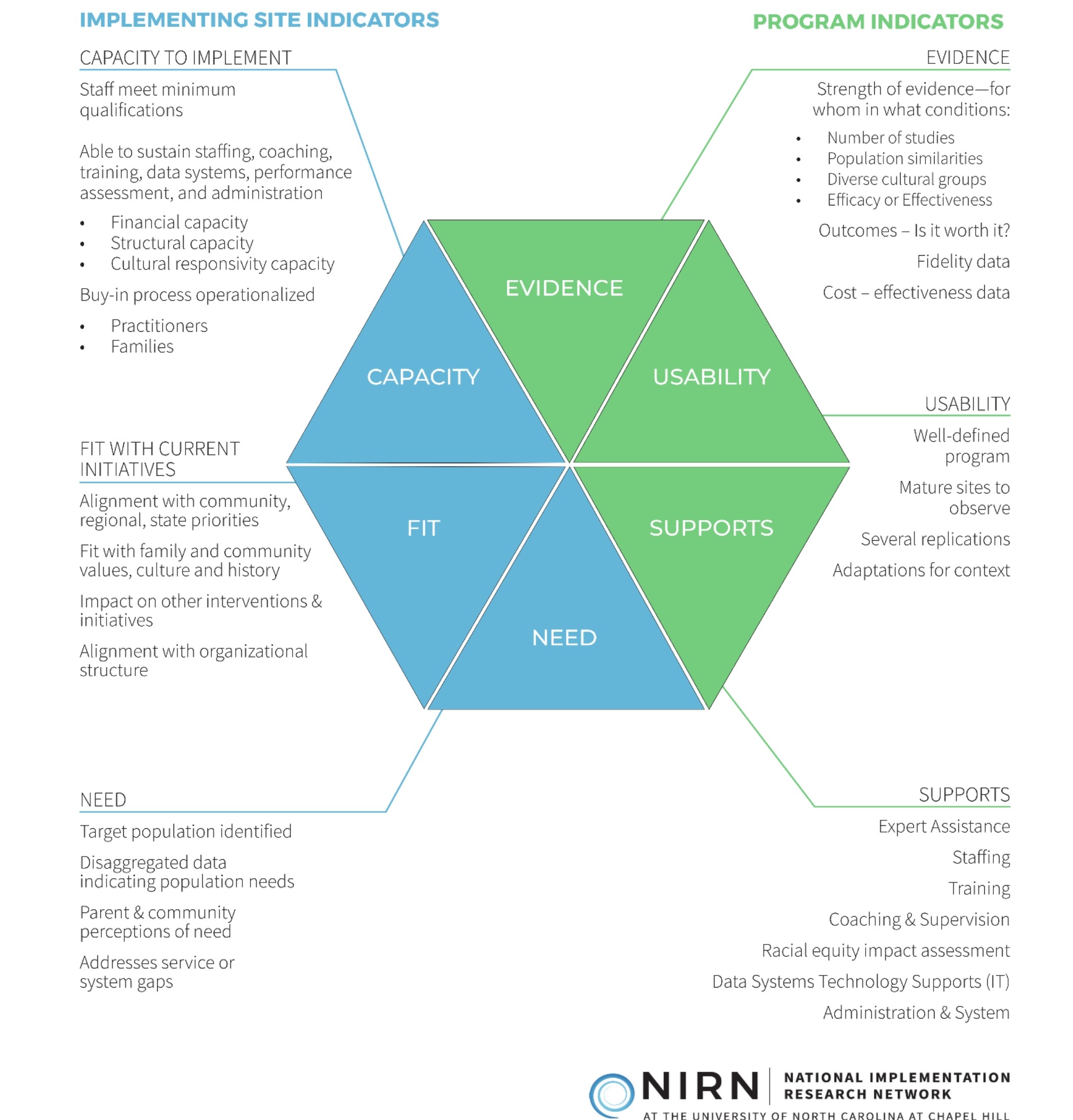

Supplement: Appendix 2 — The Hexagon Exploration Tool. [file Image_2.JPEG]
